# Non-cancer effects after proton beam therapy for pediatric tumors- a narrative review

**DOI:** 10.3389/fonc.2025.1554765

**Published:** 2025-05-30

**Authors:** Anna Zając-Grabiec, Beata Biesaga, Monika Krzyżowska, Katarzyna Drosik-Rutowicz, Justyna Miszczyk

**Affiliations:** ^1^ Department of Medical Physics, Cyclotron Centre Bronowice, Institute of Nuclear Physics Polish Academy of Sciences, Krakow, Poland; ^2^ Department of Medical Biology, Faculty of Medicine, Andrzej Frycz Modrzewski Krakow University, Krakow, Poland; ^3^ Ist Department of Radiotherapy and Chemotherapy Oncology, Maria Sklodowska-Curie National Research Institute of Oncology, Gliwice, Poland

**Keywords:** proton beam therapy (PBT), late toxicity, pediatric tumors, non-cancer effects, photon therapy (PT)

## Abstract

**Introduction:**

Radiation therapy can cause serious complications and side effects, especially in children. Proton beam therapy is considered as safer and more effective than traditional photon therapy because this type of modality offers precise radiation dose delivery to cancer cells while minimizing irradiation dose to adjacent normal tissue. Moreover, pediatric patients undergoing PBT may also experience a range of non-cancer late effects, including brainstem injury, cognitive dysfunctions, and side effects from endocrine or cardiovascular systems. The present type and frequency of non-cancer effects in children after proton therapy.

**Methods:**

Therefore, this review aims to analyze publications addressing the occurrence of side effects from proton therapy in pediatric patients, excluding those related to the induction of secondary malignancies. We used data from two publicly available databases for this review: the U.S. National Library of Medicine’s ClinicalTrials.gov (https://clinicaltrials.gov) for the analysis of clinical trials and PubMed, utilizing iCite (https://iCite.od.nih.gov)/Office of Portfolio Analysis, NIH, Bethesda, MD), a web-based application providing access to bibliometric information on publications.

**Results:**

The review of the literature shows that PBT reduces the risk of cognitive, neuroendocrine, and cardiovascular dysfunctions concerning those observed after PT. Contradictory results were observed for brain stem injury. The majority of studies found cumulative incidence (CI) of brainstem injury at a relatively low level (0.7% – 5.0%) after PBT, as compared to PT.

**Discussion:**

However, some authors underlie a higher rate of brainstem injury in children irradiated due to tumors localized in PF. Therefore, further studies, especially prospective ones, are needed to accurately describe the incidence and risk of late toxicity of proton beam therapy in children.

## Highlights

- Brain cancer stem injuries, cognitive dysfunctions, neuroendocrine and cardiovascular damage are the most frequent non-cancer late effects after proton beam therapy (PBT) in children.
- The majority of findings analyzing the risk of late toxicity after proton beam therapy in children found it decreased after PBT in relation to photon therapy (PT).
- A few studies indicated that children with tumors located in the posterior fossa (PF) or subjected to craniospinal irradiation are more susceptible to brainstem injury and cognitive dysfunctions.
- The majority of studies concerning late toxicity after proton therapy in children were conducted in small groups of patients and there are mostly retrospective studies. Further prospective studies are needed in a large group of patients, which will also allow for the analysis of factors related to radiotherapy outcomes after proton irradiation in longer follow-up studies.

## Introduction

In 2019, a total of 291,319 new cases of malignant cancers in children and 98,834 deaths due to childhood cancers were documented globally (1). The most common pediatric cancers, which also account for the majority of cancer-related deaths, include leukemia, brain and central nervous system (CNS) tumors, and non-Hodgkin lymphoma (2, 3). Generally, the cure rate for childhood cancers is approximately 85% (3). This high survival rate, particularly in developed countries, is attributed to advancements in the treatment of pediatric malignancies. Such treatment is typically a multidisciplinary approach, encompassing surgery, chemotherapy, and radiotherapy (RT) (3). Unfortunately, both the disease itself and the treatments carry the risk of long-term complications, potentially affecting all organs and systems. The severity of these complications depends on many factors like the type of cancer, its stage at diagnosis, the age at onset (children under 5 years old are particularly vulnerable), as well as the therapeutic methods used, and their intensity. The Childhood Cancer Survivor Study (CCSS) revealed that about one in five childhood cancer survivors died by the age of 30, and one in ten of these deaths was directly attributed to treatment-related factors (3, 4). Additionally, the CCSS identified specific treatment-related risk factors for late mortality, with the highest relative risk (RR) associated with radiotherapy (RR = 2.9), followed by epipodophyllotoxins (RR = 2.3) and alkylating agents (RR = 2.2) (4).

Radiotherapy is a fundamental component of treatment for many children and adolescents with malignant diseases. In pediatric patients, RT is a part of a comprehensive treatment plan and is frequently used in combination with chemotherapy and/or surgery. However, the use of RT in children requires special caution due to the increased sensitivity of developing tissues to ionizing radiation and the potential for long-term side effects in growing organisms. Secondary malignancies, neurocognitive deficits, increased risk of vascular complications such as stroke and heart disease, hormonal deficiencies, impairments in bone and soft tissue growth, vision and hearing issues, and failures in sexual and reproductive function, are among the most common late-side effects of RT in children (3, 4).

Given that the use of T in children poses a significant risk for late side effects, PBT is gaining increasing interest in the treatment of certain pediatric malignancies. Due to the Bragg peak, PBT is an advanced form of RT that allows for precise radiation dose delivery to cancer cells while minimizing exposure to normal tissues. This advantage is based on the fundamental physical principle of: its ability to control the depth at which protons release their energy within the body.

This characteristic enables physicians to precisely target tumors located deep within the body without unnecessarily irradiating surrounding normal tissues. Hence, PBT is particularly suitable for treating cancers in children, whose bodies are still growing and maturing. PBT is also preferred in childhood malignancies due to longer survivorship and a higher therapeutic ratio of protons.

PBT is divided into the modern pencil beam scanning (PBS) technique the passive scattering (PS) techniqueode (5). In this article, in addition to comparing PBT with PT, we would also like to draw attention to the determination of the type of PBT in the individual non-tumor effects after proton beam therapy for childhood cancers. However, few studies have compared the effect of PT

Nowadays, PBT is used in the treatment of pediatric brain, spinal cord, eye, soft tissue tumors, and lymphoma of the mediastinum (6). Treatment outcomes after PBT in children appear more promising compared to PT (6, 7). However, as with any medical intervention, PBT can cause potential adverse consequences and risks. In recent years, the potential for secondary tumors developing as a consequence of PBT has become an increasingly important complication. Single-center evaluations and analyses using the National Cancer Database suggest that the risk of secondary malignancies after PBT in children may be lower than with PT, especially with advanced modalities of proton therapy like pencil beam scanning that reduces neutron production (7, 8). The types of secondary cancers observed after PBT are similar to those seen after conventional radiation treatments (7–9). The most commonly reported were sarcomas, central nervous system tumors, leukemia, thyroid, and skin cancers. While the risk is reduced after PBT, it is not eliminated. Children may be at a slightly higher risk of developing secondary cancers due to their longer life expectancy after radiotherapy, which also allows more time for potential malignant transformation (10).

**Figure 1:**
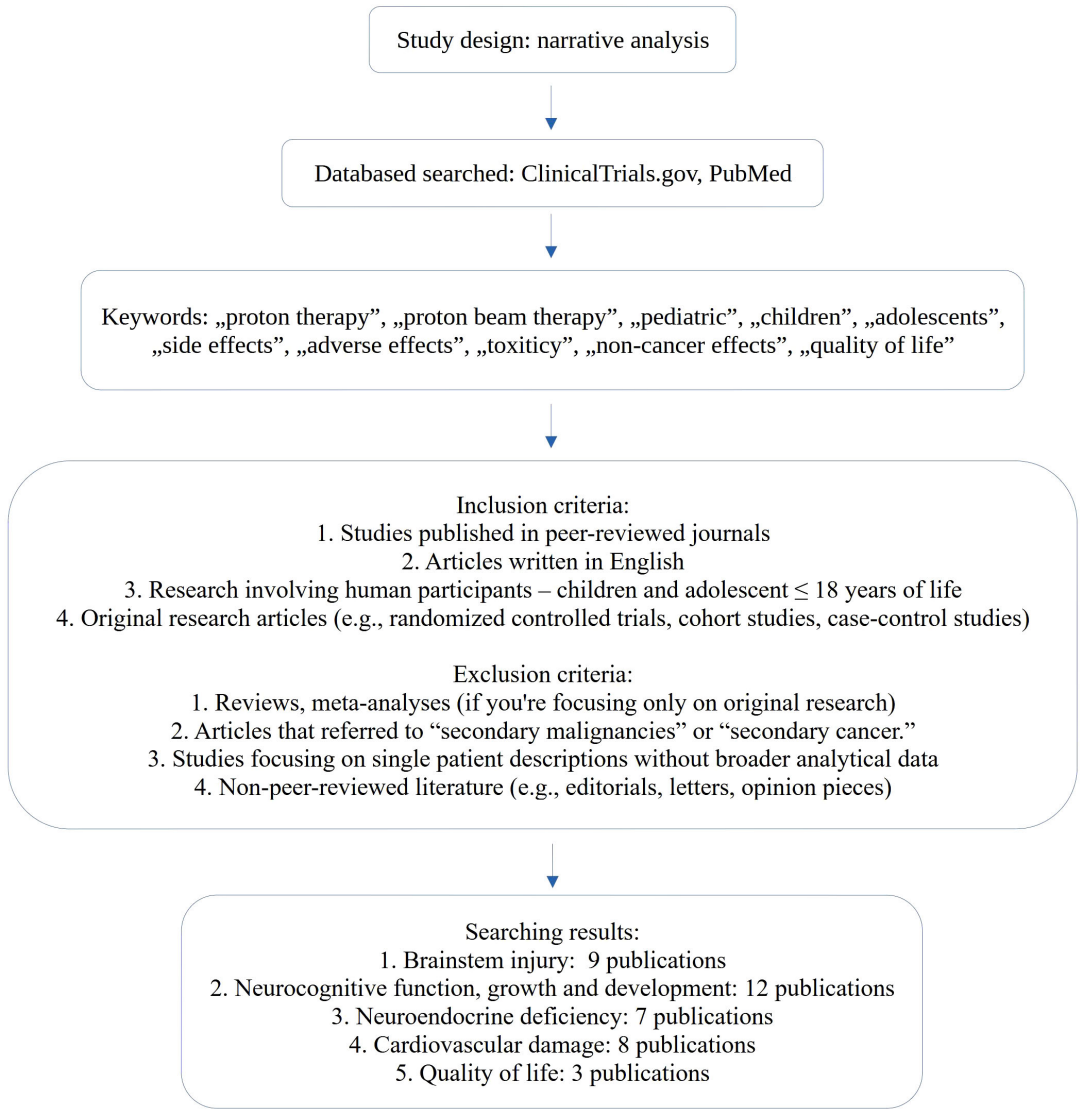
Narrative analysis.

Besides the risk of secondary malignancies, pediatric patients undergoing PBT may experience also a range of non-cancer late effects, which can vary based on factors such as the type of tumor, the patient’s age, the radiation dose, and the specific areas targeted (11). Some potential non-cancer effects that have been explored include brainstem injury, cognitive functions, and side effects from cardiovascular or endocrine systems. Our focus was to allow for a more precise and homogenous analysis, as broader radiation therapy complications inclusion would have introduced heterogeneity in both endpoints and reporting across studies. Reported outcomes vary widely depending on i.e. tumor type, treatment protocol, radiation dose, patient age, and the anatomical areas exposed to radiation. Due to the unique physical properties of proton beam therapy and its shorter clinical history compared to photon radiotherapy, long-term data collection is still ongoing, and these results should be subject to further verification. Furthermore, to our knowledge, there are only a few published articles, including one from 2020, that discussed in one paragraph the existing literature on non-cancer-related late side effects of proton therapy in children (11). Therefore, this review aims to analyze publications addressing the occurrence of side effects from proton therapy in pediatric patients, excluding those related to the induction of secondary malignancies. We used data from two publicly available databases for this review: the U.S. National Library of Medicine’s ClinicalTrials.gov (https://clinicaltrials.gov) for the analysis of clinical trials and PubMed, utilizing iCite (https://iCite.od.nih.gov)/Office of Portfolio Analysis, NIH, Bethesda, MD), a web-based application providing access to bibliometric information on publications. During the search process, we applied a combination of carefully selected keywords and Boolean operators to ensure a focused yet inclusive approach. Keywords included: *“pediatric”*, *“children”*, *“adolescents”*, *“side effects”*, *“adverse effects”*, *“toxicity”*, *“non-cancer effects”*, and *“quality of life”*. To maintain a clear scope, we excluded results related to *“secondary malignancies”* or *“secondary cancer”*. The search was conducted in English and included all articles regardless of publication year. A simplified diagram explaining the inclusion and exclusion criteria, the databases searched, the number of studies identified, and the selection process is shown on **Figure 1**.

The central nervous system tumors, alongside leukemia, represent the most common malignant neoplasms in childhood and adolescence. CNS tumors frequently arise in the PF of the skull. Their most common histological types are medulloblastoma, ependymoma, and atypical teratoid rhabdoid tumor (ATRT). Due to the frequent location of these tumors in the PF, irradiated patients are at risk of brainstem damage, potentially resulting in cranial nerve deficits, loss of motor control, impaired respiration, or even death (10, 12). Regarding photon radiation, the brainstem necrosis rate has been reported to range between 2.5% to 6.7% (12).

The present data show that PBT represents an important and preferred approach in the therapeutic management of children with brain tumors (12). The number of children receiving PBT is steadily increasing (13). Importantly, a narrative literature review concerning children after PBT suggests that the cumulative incidence (CI) of brainstem injury is relatively low (0.7% (14) – 5.0% (15)), and slightly lower compared to photon therapy (see **Table 1**). However, it is worth noting the findings of some authors (15, 19, 21), who analyzed CI or the rate of brainstem injury in children with pediatric tumors and reported significantly higher values. These findings highlight, among others, the importance of differences in linear energy transfer (LET) and consequently in relative biological effectiveness (RBE) within the spread-out Bragg peak (SOBP). Both LET and RBE are elevated at the distal ends of the SOBP (22). These observations might be elaborated by studies from Giantsoudie et al. (18). Performing a retrospective analysis of 111 children treated with PT for medulloblastoma, these researchers calculated dose and LET distribution for therapy plans (using the Monte Carlo system) and estimated RBE (based on published LET models) (18). They found higher LET levels in the subgroup of symptomatic patients with CNS injury compared to asymptomatic patients. However, no clear correlation was observed between injury sites and elevated RBE. Notable, the authors discussed limitations of the study like a small sample size and the need for further research on this topic (18).

**Table 1:** Summary of data concerning brainstem injury in children after proton therapy.

Authors	Patients	Median age [years]	Cancer Type	Type of PBT	Medium dose [Gy]	CHT	Post-PRT observation time [years]	Cumulative incidence of toxicity
Vogel et al. (2019) (14)	166	10	Astrocytoma Ependymoma	PBS	55.4	±	19.6	General - 0.7%
Haas- Kogan et al. (2018) (13)	671	5.4	Medulloblastoma – 57% Ependymoma –27%s, Glimas and ATRT – 14%	PBS	55 Gy		3.0	Grade 2+ - 2.38%. Grade 3+ - 1.3% FBI– 0.4%
Gentile et al. (2018) (16)	198	6.6	Medulloblastoma – 71.3% Ependymoma – 25.9% ATRT – 2.8%	–	54 Gy	±	4.2	General - 2.3% Medulloblastoma – 1.9% Ependymoma – 3.6% ATRT – 0.0%
Indelicato et al. (2018) (15)	179	3.5	Ependymoma (65% located in PF)	PS	54 Gy ≤3 y/o 59.4 GY >3 y/o	±	4.4	Grade2+ - 5.0% PF sites – 8.4%
Ares et al. (2016) (17)	50	2.6	Ependymoma	PBS	59.4	±	3.6	CIT – 2.8% Grade3 +-
Giantsoudi et al. (2015) (18)			Medulloblastoma	–		+	4.2	General - 3.6% Grade 3+ - 2.7%
Gunther et al. (2015) (19)	37	4.4	Ependymoma	–	59.4%	±	0,3	43%*
Indelicato et al. (2014) (20)	313	5.9	Ependymoma -23.4% Medulloblastoma – 12.1% Other - 64.5%	PS	54.0 CGE	±	2.0	General– 3.8% PE – 10.7% Grade 2 - 2.2% Grade 3 – 0.3 Grade 3- 0.3% Grade 4 – 0.6% Grade 5 – 0.3% Grade3+ – 2.1%
McGovern et al. (2014) (19)	31	1.6	ATRT	PBS	54.0 Gy – 17 pts 24 Gy – 7 pts 30,6 Gy – 7 pts	±	2.0	General - 16%* PF – 9.7%

ATRT, atypical teratoid/rhabdoid tumors; CHT, chemotherapy; CIT, cumulative incidence of toxicity; FBI, fatal brainstem injury; PF, posterior fossa; PBS, PS.

Currently, LET and RBE variability within the SOBP are not considered in proton therapy planning (13, 18). Therefore, more conservative approaches to brainstem irradiation are being emphasized (13). For this reason, the Children’s Oncology Group has introduced changes to the ACNS0831 protocol for ependymoma, imposing stricter proton dose constraints on the brainstem (13) (see **Table 1**). In contrast to the aforementioned results regarding the CI of brainstem injury, other authors have reported much lower CI rates (0.7% – 2.3%) in groups of children with primary PF tumors (13, 14, 16, 17). Several factors influence the development of brainstem injury, including total proton dose, irradiated brainstem volume, adjuvant chemotherapy treatment, and the interval between surgery and RT (13–17, 19, 21). Regarding dose and irradiated volume, Gentile et al. (16) found that among five patients with brainstem injury after PBT, four had a brainstem in the highest dose quartile (>55.8 Gy), and the V_55_ volume in the highest tertile (>6.0). On the contrary, Gunther et al. (19) reported a higher median D_50_ (≥54 Gy RBE) for patients with radiographic changes compared to those without them. Some studies have also shown that adjuvant chemotherapy (16, 18, 21) is associated with an increased risk of brainstem injury, likely due to its radiosensitizing effect (23). This result may be explained by insufficient tissue healing following resection. However, the effect of patient age on the risk of PBT-induced brainstem injury remains unclear. Some studies identify younger age as a risk factor (19, 20), while others suggest that older age (>5 years (16)) at diagnosis is associated with a higher incidence of imaging changes. It is also important to consider the varied definitions of brainstem injury used in reviewed studies. Some authors define this effect based on MRI changes coupled with new neurological symptoms unrelated to tumor progression (16, 18), while others include only MRI using previously published scales (19), or the CTCAE scale (13, 15, 20). an overestimation of clinically significant injury, as some imaging abnormalities might be transient, subclinical, or unrelated to functional impairment. In contrast, MRI diagnostics enable differential diagnosis of changes in nervous tissue resulting from either active neoplastic infiltration or additionally, intrinsic radiation sensitivity likely plays a role, although little is known about genetic or other factors influencing increased pediatric brainstem injury related to PBT (see **Table 1**).

To summarize the data on the incidence of brainstem injuries in children after PBT, further studies, especially prospective ones are needed to precisely describe the incidence and risk of brainstem necrosis in children. The PBS. At the same time, it is necessary to ensure that the risk of recurrence is not increased in the photon and proton cohorts with a longer period of clinical and imaging follow up.

## Neurocognitive dysfunctions

Children treated with cranial radiation therapy for brain tumors are at an increased risk of neurocognitive impairment, affecting both overall intellectual functioning (expressed, for example, by full-scale IQ - FSIQ) and specific cognitive domains such as executive functions, attention, memory, processing speed, and control (24). Preclinical studies have identified white matter and hippocampal substructures as critical areas involved in radiation-induced cognitive impairment (25). For decades, the late cognitive and academic effects observed in patients treated with conventional photon-based radiotherapy have been studied. Following this type of RT, declines in global IQ1–3 and specific cognitive domains (e.g., executive functions, attention, language, and fine motor control) have been commonly reported (26). Furthermore, survivors experience poorer academic performance, particularly in academic fluency (i.e., the ability to quickly perform basic tasks in reading, writing, and mathematics) compared to their peers. However, it remains unclear whether newer approaches, such as proton radiotherapy, pose an increased risk of neurocognitive impairment. A potential advantage of PBT over PT is its ability to reduce the exposure of healthy tissue surrounding the target area, potentially mitigating its harmful effects on neurocognitive outcomes. In line with this observation, most studies [27-35] have not demonstrated an increased incidence of neurocognitive impairment (see **Table 2**). However, the Child et al. publication (29) analyzing a total of 88 children who underwent PBT (58 patients) or PT (30 patients) reported a decline in neurocognitive function in all cognitive domains assessed after RT. All groups were significantly below the population mean on processing speed and motor coordination. On all other cognitive measures, the PBT focal group did not differ significantly from the population mean. In contrast, the PT group scored significantly below the population mean on all cognitive measures except for the attention tasks. Both the PT and PBT groups scored significantly below the population mean on the FSIQ. In terms of academic proficiency, all groups scored significantly below the standards on measures of mathematical fluency and writing (most p < 0.01). The PT group performed worse than the PBT group on cognitive and academic measures (29).

**Table 2:** The potential non-cancer effects of proton therapy, such as neurocognitive function, growth and development, and quality of life in pediatric patients.

Author	Patients	PRT initiation age [yrs]	Cancer type	Type of PBT	Medium dose [Gy]	CRT	Post-PRT observation time [yrs]	Incidence of neurocognitive impairment
Mash et al. (2023) (27)	12	4.6	Low-Grade Glioma Embryonal tumor Ependymoma	–	53.55	±	8.9	No incidence of neurocognitive impairment: No changes in white matter integrity.
Ali et al. (2021) (28)	41	2.0	Glioma-42.3% Medulloblastoma/ PNET-7.7% Ependymoma-19.2% Atypical teratoid rhabdoid tumor Primitive neuroectodermal tumor Germinoma – 11.52% Other-15.4%	–	nd	+	2.3	No incidence of neurocognitive impairment
Child et al. (2021) (29)	58	7.7	Glioma-25.9% Medulloblastoma/ PNET-32.7% Ependymoma-13.9% Germ Cell Tumor-17.2% Other-10.3%	PS	F: 50.4 CSI: 54.0	nd	6.1	Variable - F *vs* CSI: FSIQ - 13.3% *vs* 28.6%) VCI - 3.3% *vs* 28.6% PRI – 10% *vs* 17.9%), WMI - 10% (F), 17.9% PSI – 30% *vs* 46.4% Fine Motor – 26.7% *vs* 60.7 Switching (Verbal) - 26.7% *vs* 17.9% Switching (Graphomotor) – 30% *vs* 46.4% Inhibition/Switching – 23.3% *vs* 32.1% Verbal Learning – 20% *vs* 32.1% Verbal Memory *vs*16.7% *vs* 21.4% Visual Learning 10% *vs* 25% Visual Memory 13.3% *vs* 14.3% Attention 13.3% *vs* 17.9% Omissions 3.3% *vs* 10.7% Reading fluency 23.3% *vs* 42.9% Writing fluency 26.7% *vs* 53.6% Math Fluency 33.3% *vs* 42.9%
Weusthof et al. (2021) (30)	26	9.4	Glioma-42.3% Medulloblastoma/ PNET-7.7% Ependymoma-19.2% Craniopharyngioma – 3.8* Germinoma – 11.52% Other-15.4%	–	51.3	±	3.5	No incidence of neurocognitive impairment
Yip et al. (2020) (31)	14		Medulloblastoma – 36.0%	–	nd	nd	4.3	No incidence of neurocognitive impairment
Kahalley et al. (2020) (32)	37	8.6	Medulloblastoma	PBS	54.0		10	No incidence of neurocognitive impairment
Gross et al. (2019) (33)	58	7.4	Craniopharyngioma - 8.6% Medulloblastoma/PNET - 44.8% Ependymoma –8.6% Germinoma -15.5% Glioma -15.5% Other - 6.9%	–	nd	±	2.6	No incidence of neurocognitive impairment
Peterson et al. (2019) (34)	22	10.0	nd	–	nd	nd	nd	No incidence of neurocognitive impairment
Yang et al. (2018) (35)	4	5.9	Atypical meningioma Retinoblastoma Ependymoma	PBS	nd	+	nd	No incidence of neurocognitive impairment
Pulsifer et al. (2018) (36)	60	12.3	Medulloblastoma – 38.3% Gliomas – 18.3% Craniopharyngioma – 15.0% Ependymoma – 11.7% Other – 16.7%	PBS	52.2	±	3.6	FSIQ – 40% Processing Speed – 35%
Kahalley et al. (2016) (37)	90	9.2	Glioma – 22.2% Medulloblastoma/PNET – 37.8% Ependymoma – 4.4% Germ cell tumor – 18.9% Other – 16.7%	90% PS 10% PBS	54		5	No incidence of neurocognitive impairment

CRT, conformal radiotherapy; CSI, craniospinal irradiation; F, focal; FSIQ, Full Scale Intelligence Quotient; nd, no data; PRI, Perceptual Reasoning Index; PSI, Processing Speed Index; VCI, Verbal Comprehension Index; WMI, Working Memory Index.; PBS, PS.

Overall, the results indicated that patients who received focal PBT achieved results that were within the norm on most cognitive and academic measures compared to children who received PT. The results of patients who received PBT were generally comparable to normative results for typically developing children. Even the weaknesses in processing speed, fine motor skills, and academic abilities fell slightly below the average range, indicating clinically mild challenges in these areas for this group. Similarly, Pulsifer et al. (36) demonstrated that patients under 6 years of age and those receiving craniospinal irradiation (CSI) were particularly vulnerable to IQ loss. Importantly, adaptive functioning did not worsen, and processing speed remained within normal limits (i.e., standard score ≥ 90) in both the focal and CSI PBT groups (36).

Several studies have also directly compared neurocognitive function following PBT and PT (see **Table 3**). These comparisons indicated that the subgroup of children receiving CSI PT was associated with the highest risk of neurocognitive decline. In this subgroup, particularly low cognitive functioning was observed, with 76% of individuals showing significant reductions in global intellectual functioning, and 53–88% experiencing difficulties across all tasks related to cognitive and academic fluency (except for a computerized attention task). The CSI PBT group also demonstrated lower scores in overall intellectual functioning and cognitive domains sensitive to radiotherapy (e.g. working memory, processing speed, fine motor skills, executive functions, and memory). Nonetheless, a smaller percentage of this group fell into the impaired range compared to the CSI PT (38). Pediatric patients with brain tumors who received PBRT scored significantly higher on most of the neurocognitive outcomes than those who received XRT (38).

**Table 3:** Comparison of children’s neurocognitive dysfunction between proton beam therapy and photon irradiation.

RT				PBT		
Authors	Patients	Total radiation dose [Gy]	Main results	Patients	Total radiation dose [Gy]	Main results
Mash et al. (2023) (27)	10	T: 53.4	Reduction of white matter integrity Relative significantly lower of cognitive and motor functions (FSIQ, VCI, PRI, WMI) F group: relative significantly lower overall intelligence, verbal reasoning, visual-motor skills, motor coordination	12	53.5	No reduction of white matter integrity. No decrease in cognitive and motor functions (FSIQ, VCI, PRI, WMI) F group: no change in respect to overall intelligence, verbal reasoning, visual-motor skills, motor coordination
Child et al. (2021) (29)	30	F: 54.0 CSI: 54.0	F group: significant decrease concerning FSIQ, verbal and graphomotor switching, math, reading and writing fluency, processing spin, fine motor coordination CSI: significant impairments in motor skills, processing speed, attention and interpersonal relations, math, reading and writing fluency, processing spin, fine motor coordination	58	F: 50.4 CSI: 54.0	F group: math and writing fluency, processing spin, fine motor coordination CSI: significant decrease concerning FSIQ, verbal and graphomotor switching, math, reading and writing fluency, processing spin, fine motor coordination
Kahalley et al. (2020) (32)	42	T: 55.8 CSI: 23.4	Significant decrease in global IQ, working memory, and processing speed	37	T: 54.0 CSI: 23.4	Stable intellectual outcomes in most domains (IQ, perceptual reasoning, working memory),(even in the context of CSI) Processing speed: decrease over time
Gross et al. (2019) (33)	67	F: CSI:	Relative lower full-scale IQ and processing speed, higher verbal IQ, and general adaptive function F group: relatively lower PSI CSI: relative lower VIQ, FSIQ/GAI	58	F: CSI:	Relative higher full-scale IQ and processing speed, higher verbal IQ, and general adaptive functions F group: relatively higher PSI CSI: relative higher VIQ, FSIQ/GAI
Pulsifer et al. (2018) (36)	60		Processing speed and working memory skills were significantly lower at follow-up for patients treated with CSI, regardless of age.	90	52.2	Motor, social interaction, personal living, community living - no significant change in adaptive functioning was found after PRT, regardless of age or radiation field
Kahalley et al. (2016) (37)	60	T: 54.0 CSI: 23.4	IQ decreased by 1.1 points per year in the RT group. IQ was lower in the RT group (by 12.5 points) compared to the PBT group.	90	T: 54.0 CSI: 23.4	No change in IQ over time.

CSI, craniospinal irradiation; F, focal; FSIQ, Full Scale Intelligence Quotient; GAI, General Ability Index; PRI, Perceptual Reasoning Index; PSI, Processing Speed Index; T, total; VCI, Verbal Comprehension Index; WMI, Working Memory Index.

It is also important to note that many factors, both related and unrelated to radiotherapy, influence neurocognitive outcomes. During RT, particular attention should be paid to the percentage of CSI use and the increased dose administered to specific brain regions such as the temporal lobes, hippocampus, and frontal lobes, as these areas may have a more detrimental effect on cognitive and social functioning compared to other brain regions (39, 40). In the study by Kahalley et al. (37), the majority of patients treated with PB. Additionally, the longer follow-up period available for photon-treated patients should be considered (33, 37). Other factors include chemotherapy exposure (41) and age at diagnosis (22, 24, 26, 42).

Non-treatment-related factors include pre-existing comorbidities (such as seizure disorders, stroke, hydrocephalus, or the need for VP shunts) and genetic factors. It has also been demonstrated that chemotherapy and surgery may have a potential negative impact on neurocognitive outcomes independently of radiotherapy (43, 44). Furthermore, attention should be given to the tests measuring overall neurocognitive abilities, such as processing speed (an index assessed in Wechsler-based evaluations like WISC-IV and WAIS-IV IQ). Although these parameters are most affected by radiotherapy, they are not considered in abbreviated IQ tests such as Estimated IQ, WASI-II, or the General Ability Index. Therefore, some authors recommend avoiding these tests in prospective studies to prevent the potential underestimation of cognitive decline (11).

## Neuroendocrine dysfunctions

Neuroendocrine dysfunctions, alongside neurocognitive impairment, are one of the most frequently reported late effects of radiation therapy in children treated for brain tumors. These dysfunctions are directly related to damage to the hypothalamic-pituitary axis (HPA). CSI, through its impact on the HPA, can also affect organs beyond the central nervous system (CNS), including the thyroid, heart, lungs, liver, pancreas, kidneys, gonads, and bones, including spinal growth abnormalities (45). Therefore, children receiving CSI are at risk of multiple endocrinopathies, including growth hormone deficiency (GHD), hypothyroidism, adrenal insufficiency, and abnormal sex hormone production manifesting as hypogonadism or precocious puberty. These long-term deficiencies are a significant cause of morbidity among brain tumor survivors, affecting up to 80% of this population and being associated with an increased risk of various other medical conditions, necessitating chronic treatment and leading to high healthcare costs. The extent of radiation-induced endocrinopathies appears to be dose-dependent (46). The most common endocrinopathies are considered to be growth hormone deficiency and hypothyroidism. In the cohort treated with photon irradiation, the prevalence of adrenal insufficiency, precocious puberty, and sex hormone deficiency was 8%, 16%, and 19%, while for it was 5%, 18% and 3%, respectively (47).

Proton beam radiotherapy reduces radiation exposure to normal tissues, such as the hypothalamus and pituitary gland (10). Theoretically, this type of radiation method may prevent the development of neuroendocrine dysfunction. Multiple dosimetric comparative studies have demonstrated the potential to reduce the radiation dose received by the HPA when compared to 3D photon therapy or intensity-modulated photon therapy (48, 49). Based on PBT dosimetric advantage, modeling studies have suggested that proton therapy compared to photon therapy is associated with a reduced risk of late endocrinological effects (50).

A narrative literature review has identified that PBT was also associated with an increased risk of neuroendocrine dysfunction (see **Table 4**). In the cited studies, the incidence of growth hormone deficiency (GHD) ranged from 37.5% (51) to 60% (55). However, the lowest percentage observed in the analysis performed by Yip et al. (51) was noticed for the entire cohort included in the study, regardless of whether the children received CSI or not. Importantly, the follow-up period was 4.4 years. Of note, most endocrinopathies can manifest within the subsequent 6 years following cancer treatment, as endocrine complications have been reported decades later (58). Therefore, the incidence of endocrine disorders in patients with a follow-up period of less than 5 years may be underestimated. Another frequently occurring neuroendocrine dysfunction after PBT is hypothyroidism, with the incidence ranging between 17.7% (51) to 47.5% (55). Other neuroendocrinopathies were reported at significantly lower rates. Aldrich et al. (52) in univariate analyses showed no clinical or demographic factors to be associated with the occurrence of any endocrinopathy, except for moderate differences in GHD between treatment protocols (see **Table 4**).

**Table 4:** The narrative review of endocrine deficiency after proton beam therapy in children.

Author	Patients	PBT initiation age [yrs]	Cancer type	Medium dose [Gy]	CRT	Post-PBT observation time [yrs]	Incidence of endocrine deficiency
Yip et. al. (2022) (51)	32		Medulloblastoma - 38%	54.0	±	4.4	Growth hormone deficiency – 37.5% *vs* 50.0% (CSI) Hypothyroidism - 19%. *vs* 17.7% (CSI) Sex Hormone Deficiency- 6.3% vs 0.0% (CSI) Hormone Replacement Therapy 37.5% vs 50.0% (CSI)
Aldrich et al. (2021) (52)	64	7.6	Medulloblastoma	CSI< 30 Gy- 63.5 (n=40) CSI≥30 (n=23)	nd	5.6	Primary Hypothyroidism- 28% Growth hormone deficiency – 52.5% Adrenal insufficiency- 5% Endocrine replacement therapy- 55.0% Sex Hormone Deficiency-2.5% Precocious puberty- 17.5%
Bielamowicz et al. (2018) (53)	41		Meduloblastoma		nd	3.8	Hypothyroidism – 19.0% Primary hypothyroidism - 7.3% Central hypothyroidism – 9.8%
Eaton et al. (2016) (54)	40	6.2	Medulloblastoma	TB – 60 (n=24) PF – 30 (n=12) PF → TBa – 10 (n = 4)	+	5.8	Hypothyroidism- 22.5% Growth hormone deficiency- 52.5% Adrenal insufficiency- 5% Sex Hormone Deficiency-2.5% Precocious puberty- 17.5%
Greenberger et al. (2014) (55)	29	11.0	Low-grade gliomas of the brain or spinal cordOther-15.4%	52.2	±	7.6	Growth hormone deficiency – 60.0% Hypothyroidism – 47.5% Cortisol insufficiency – 22.5% Testosterone deficiency – 16.0% Elevated prolactin – 12.5% Diabetes Insipidus – 9.0% Precocious Puberty – 6.0%
Viswanathan et al. (2011) (56)	31	11.9	Craniopharyngioma - 7 Medulloblastoma - 6 Glioma- 4 Other – 14	F: 50.4 CSI: 54.0	±	1.8	Growth hormone deficiency (n = 6), TSH deficiency (n = 4), ACTH deficiency (n = 4), and hypogonadotropic hypogonadism (n = 4).
Yinuo Li et al. (2023) (57)	11	8	Rhabdomyosarcoma - 2 Neuroblastoma- 8 Osteosarcoma- 1		±	2,04	The median relative change in irradiated kidney volume was 16.42% compared to the control group after 1 year.

CRT, conformal radiotherapy; CSI, craniospinal irradiation; nd, no data.

Several studies have directly compared the incidence of neuroendocrine dysfunctions between PBT and PT. Yip et al. (51) found that proton therapy is associated with a lower risk of hypothyroidism (29% for PT vs. 19% for PB

Yinuo Li et al. (57) analyzed the late effects of proton therapy (PBT) in children with malignant tumors. The kidney is frequently irradiated in radiotherapy for childhood malignant tumors, such as childhood neuroblastoma and rhabdomyosarcoma. In all cases, the kidney was irradiated through the primary lesion. In the irradiated and contralateral control kidneys, the median volume changes were 5.63 and 5.23 mL/year; and the median % volume changes at 1 year were 8.55% and 9.53%, respectively. The median relative volume change of the irradiated kidneys at 1 year was 16.42% compared with the control kidneys. The larger the irradiated volume, the greater the loss of renal volume. The volume reduction was significantly greater in patients aged 4–7 years than in patients aged 2–3 years. The results suggest that kidneys exposed to PBT for the treatment of childhood malignancies show continued atrophy during follow-up. The degree of atrophy increases with increasing radiation dose, larger irradiated volume, and older age. However, with growth and maturation, the contralateral kidney becomes progressively larger and less radiosensitive (57).

Summarized, reviewed studies support the observation that proton therapy is associated with a lower incidence of hypothyroidism, thyroid protection, and sex hormone deficiency compared to conventional X-ray therapy. These findings highlight the potential benefits of PBT application, especially in minimizing endocrine sequelae in patients undergoing treatment for medulloblastoma. Further investigation into growth hormone deficiency and non-hormonal growth changes in patients treated with both protons and photons is necessary to establish comprehensive treatment protocols.

## Cardiovascular damage

Increased cardiovascular morbidity and mortality are well-documented late toxicities following mediastinal radiotherapy in patients with Hodgkin lymphoma (HL) (59). Although HL is a rare malignancy in the general population, a significant percentage of cases occur in adolescents and young adults (60), which makes it the most common malignancy among individuals aged 15 to 19 years. Hodgkin lymphoma is characterized by a high probability of long-term survival, which allows sufficient time for latent radiation-induced damage to manifest, ultimately affecting both quality of life and, in some cases, life expectancy. Cardiovascular disease (CVD) is viewed as the most common non-malignant cause of death among HL survivors (61).

RT targeting the cranial or craniospinal regions for brain tumors or leukemia can damage the hypothalamic-pituitary-thyroid axis, especially with doses exceeding 20 Gy (62–64). This damage may disrupt metabolic processes and hormone regulation, thereby increasing CVD risk factors such as obesity, dyslipidemia, insulin resistance, and diabetes (63–66). Chemotherapy agents, particularly anthracyclines, cyclophosphamide, cytarabine, cisplatin, and ifosfamide, often administered in combination with radiotherapy, can also adversely affect the cardiovascular system by impairing myocardial function or causing peripheral damage (64). Post-radiation myocardial toxicity is associated with diffuse interstitial fibrosis, microvascular damage, and valvular fibrosis (64). In the vascular system, chronic inflammation induced by radiation has increased the risk of atherosclerosis development (64, 65).

Studies evaluating whole-heart dosimetric parameters concerning late cardiotoxicity have shown that increased cardiotoxicity correlates with higher whole-heart dose, greater intracardiac dose inhomogeneity, male sex, and increasing age (65–67). As a result, hematologists and oncologists may accept higher relapse rates and salvage therapies in exchange for omitting radiotherapy to reduce late toxicities (68). With the growing number of proton therapy centers, more young HL patients have received PBT (69). Several studies have compared cardiovascular toxicity following proton beam therapy and photon radiotherapy. Zhang et al. (70) analyzed 17 pediatric patients with medulloblastoma treated with either passively scattered protons (PS) therapy or craniospinal irradiation using field-in-field photons. They compared the risk of lifetime attributable risk (RLAR) and relative risk (RRs had a significantly higher RLAR for cancer mortality than boys. In earlier work published by the same authors (71), comparisons of cardiac toxicity risks in pediatric patients with Hodgkin’s disease (HD) and medulloblastoma (MB) showed that PS therapy reduced predicted cardiac toxicity risks compared to photon therapy, particularly in the MB patient cohort. Hoppe et al. (72) conducted a study on 13 pediatric and adolescent HL patients, comparing three-dimensional conformal radiotherapy (3DCRT), intensity-modulated radiotherapy (IMRT), and proton therapy (PBT) for involved node radiotherapy (INRT). The authors found that proton therapy significantly reduced average heart doses compared to 3DCRT and IMRT, lowering radiation exposure to all major heart subunits. Consequently, as the authors suggested proton therapy reduced the risk of cardiac toxicity. Similarly, Lautenschlaeger et al. (73) showed that in a cohort of young adult HL patients, PBT provided significant dose-sparing benefits to the lungs, coronary arteries, and heart valves compared to photon-based plans.

Summarized, these studies suggest that proton vs. photon therapy may reduce the risk of cardiac toxicity and secondary cancer incidence in pediatric patients with Hodgkin’s lymphoma and medulloblastoma. The extent of these benefits is influenced by many factors including cancer type, patient age, and specific treatment protocols.

## Quality of life

The Childhood Cancer Survivor Study found that among young adult survivors of childhood cancer diagnosed between 1970 and 1986, at least 1 of 6 health status domains (general health, mental health, functional status, activity limitations, cancer-related pain, and cancer-related anxiety) deteriorated moderately or severely in 44% (74). Currently, the cumulative incidence of chronic disease recorded 30 years after cancer diagnosis is 73%, with a cumulative incidence of 42% for severe, disabling, or life-threatening conditions or death attributable to chronic disease (75). Concerning PBT, Garcia-Marqueta et al. (76) evaluated the quality of life in a group of 207 patients with intracranial meningioma treated with pencil-beam scanning proton beam therapy proton therapy was assessed using the PEDQOL questionnaires, evaluating physical, emotional, social, and school functioning domains. The study demonstrated an estimated 5-year local control and overall survival rates of 19.4% and 100.0%, respectively. Except for one patient who developed a cataract requiring surgery, no grade ≥3 late toxicities were reported. During the first year after PBT, one child required educational support, one needed to attend a special school, one had social difficulties, and three children required assistance with daily basic activities (DBA). Three years post-PBT, only one child continued to require assistance for DBA. Proton therapy, delivered mode BT therefore has a clear advantage in the treatment of brain tumors, especially in children. In fact, an improvement in neuropsychological outcomes has been observed in pediatric patients with brain tumors after PBT (76).

There are also studies comparing health-related quality of life (HRQoL) after PBT and PT. These studies assessed the parameter known as health-related quality of life (HRQoL). Kaltahau et al. (77) investigated HRQoL in a group of 142 pediatric patients aged 2–18 years with intracranial tumors treated with proton radiotherapy at Massachusetts General Hospital, followed for six years post-treatment. The authors demonstrated a significant correlation between lower full-scale IQ (FSIQ) and poorer HRQoL outcomes. Additionally, the use of craniospinal irradiation (CSI) and chemotherapy was associated with worse HRQoL outcomes. A study performed by Yock et al. (78) focused on comparing HRQoL outcomes in this patient group as reported by parent-proxies. Three years after treatment, the proton cohort scored 10 points higher in the overall baseline HRQoL score, and this difference was statistically significant (78). This prospective study of children with brain tumors treated with PBT shows the influence of disease type and treatment intensity on HRQoL. Worse HRQoL scores were 19 shown in the domains of anxiety, communication, and worry, suggesting that increased support from psychiatrists, psychologists, and medical staff may also improve HRQoL scores.

Results of the above-mentioned studies emphasize the long-term benefits of PBT in reducing late toxicities and improving QoL outcomes in pediatric oncology patients.

## Summary, conclusions, and future directions

Proton radiotherapy is considered an effective and precise cancer treatment method causing minimal side effects. We conducted a narrative review of the published literature on the non-cancer effects after PBT, but we did not perform a meta-analysis due to considerable variability between studies. In published articles, there are multiple sources of heterogeneity, some of which include differences in cancer types, population characteristics, study methodologies, type of radiotherapy used, and fractionation schedule. radiotherapy (79–81). Compared to photons, the LET which is the predominant factor that influences the RBE increases rapidly with depth along the SOBP reaching a maximum value at the distal edge of the Bragg peak (82). This potentially can increase the radiation DNA damage to healthy tissue and may influence outcomes. Several pre-clinical and clinical studies have investigated the LET and RBE distributions, however, much is still unknown about the mechanism by which LET affects RBE for healthy tissue compared to cancer cells (82). In a recently published study, the authors report on the association of LET and dose which may contribute to greater radiation risk of necrosis after pencil beam scanning proton therapy in 33 pediatric patients with posterior fossa tumors (83). On the other hand, in 36 pediatric brain tumor patients treated with passive scattering proton therapy, the authors highlighted that the elevated LET could be a minor contributor to the observed brainstem toxicity, but a very minor trend towards higher LET and increased RBE-weighted dose was seen in patients with brainstem toxicity (84). Therefore, the individual assessment of LET and RBE in preclinical and clinical studies for pediatric tumors should be explored further.

The other sources of heterogeneity that limit the presented review studies are the applied two alternative modes of PT delivery. for highly conformal dose distribution, PS (85). The clinical significance for pediatrics of the differences between the two alternative proton modes is not well understood. The information about treatment modes like PBS or PS has been identified in our review but future studies will be necessary to better compare the two treatment modalities on treatment outcomes after proton beam therapy for pediatric tumors.

Limitations of the narrative review also include the exclusion of unpublished manuscripts and abstracts from conference proceedings.

The majority of findings analyzing the risk of late toxicity after proton beam therapy in children found a decrease in this risk after PBT in relation to. The analysis indicates that PBT generally reduces the risks of cognitive, neuroendocrine, and cardiovascular complications compared to conventional In the study by Michael T et al. (86), proton beam therapy has been shown to be the preferred radiotherapy modality for childhood cancers, which are rare and heterogeneous diseases. Radiation to the head and neck region is associated with a range of radiotherapy complications affecting vision, hearing, feeding, and growth. Support for proton therapy comes from risk modeling and a limited number of cohort series (86). We compared the efficacy and expected toxicity of proton and photon radiotherapy for childhood cancers and examined the benefits of proton radiotherapy in reducing acute and late radiation toxicities, including the risk of secondary malignancies, vision, and cognition. Proton therapy demonstrated few acute and late radiotherapy toxicities and provided similar rates of locoregional control in pediatric patients with head and neck cancer. In addition, Masashi Mizumoto et al. (87) valuated the long-term benefits of PBT in cancer survivors. Retrospective observational study of 62 pediatric patients who received PBT for 5 or more years. Analysis showed that the irradiated site (head and neck, brain) was significantly associated with late toxicities. No malignant secondary tumors occurred in the irradiated field. Data suggest that PBT has the potential to reduce the risk of late mortality and secondary malignancy (87). However, even with its advantages, non-cancer effects can arise, which vary depending on the treatment site, dose, and age of the child.

Our findings generally support a lower incidence of certain late toxicities following PBT in comparison to photon therapy, especially in pediatric patients. However, the evidence remains inconclusive in several key areas. Some studies suggest that children treated for posterior fossa tumors or undergoing craniospinal irradiation may still be at risk of brainstem injury or neurocognitive decline. Moreover, many published reports are retrospective, based on small sample sizes, and suffer from inconsistent definitions and reporting of toxicity outcomes.

Different childhood cancers exhibit varying degrees of radiosensitivity and associated risk of late toxicity. The above studies have shown that patients with medulloblastoma treated with PBT had cognitive deficits and endocrine dysfunction compared with photon therapy. Patients with ependymoma treated with PBT have comparable tumor control with potentially fewer neurocognitive side effects, similar to children with rhabdomyosarcoma. s can be seen in **Tables 1**–**4**, age at the time of treatment seems to be a factor influencing susceptibility to radiation-induced side effects. Children under 5 years of age are particularly susceptible to neurocognitive disorders due to ongoing brain development. Analysis may provide a more structured and comprehensive understanding of the effects of PBT in different pediatric populations. This stratified approach may also guide future research and clinical decision-making by ensuring that treatment protocols and of each subgroup.

Given these limitations, it is evident that future research must move beyond retrospective analyses and isolated institutional experiences. There is a pressing need for large-scale, prospective studies conducted across multiple centers, with harmonized methodologies and long-term follow-up. Such studies should not only assess clinical endpoints but also include comprehensive evaluations of patient-reported outcomes and neuropsychological functioning, particularly in pediatric populations where subtle cognitive deficits may emerge years after treatment. Furthermore, the role of biological and treatment-related modifiers—such as age at exposure, anatomical site, and individual radiosensitivity—requires further exploration to identify patients at higher risk of adverse effects.

Another critical direction for future studies is the comparative evaluation of proton and photon therapies through controlled clinical trials, where feasible, or well-designed observational studies employing matched cohorts and robust statistical methods. These investigations should focus not only on dosimetric advantages but also on long-term functional outcomes and quality of life. At the same time, international collaboration and the creation of shared databases or registries could greatly enhance the power and generalizability of findings, allowing researchers to pool data and identify meaningful patterns that may not be apparent in single-center studies. Additionally, the incorporation of novel biomarkers—including imaging-based and molecular indicators—holds promise for more precise risk stratification and individualized treatment planning. As the field of radiogenomics evolves, future research should aim to integrate these tools into clinical protocols to better predict and mitigate the risk of late toxicity.

**Figure 2:**
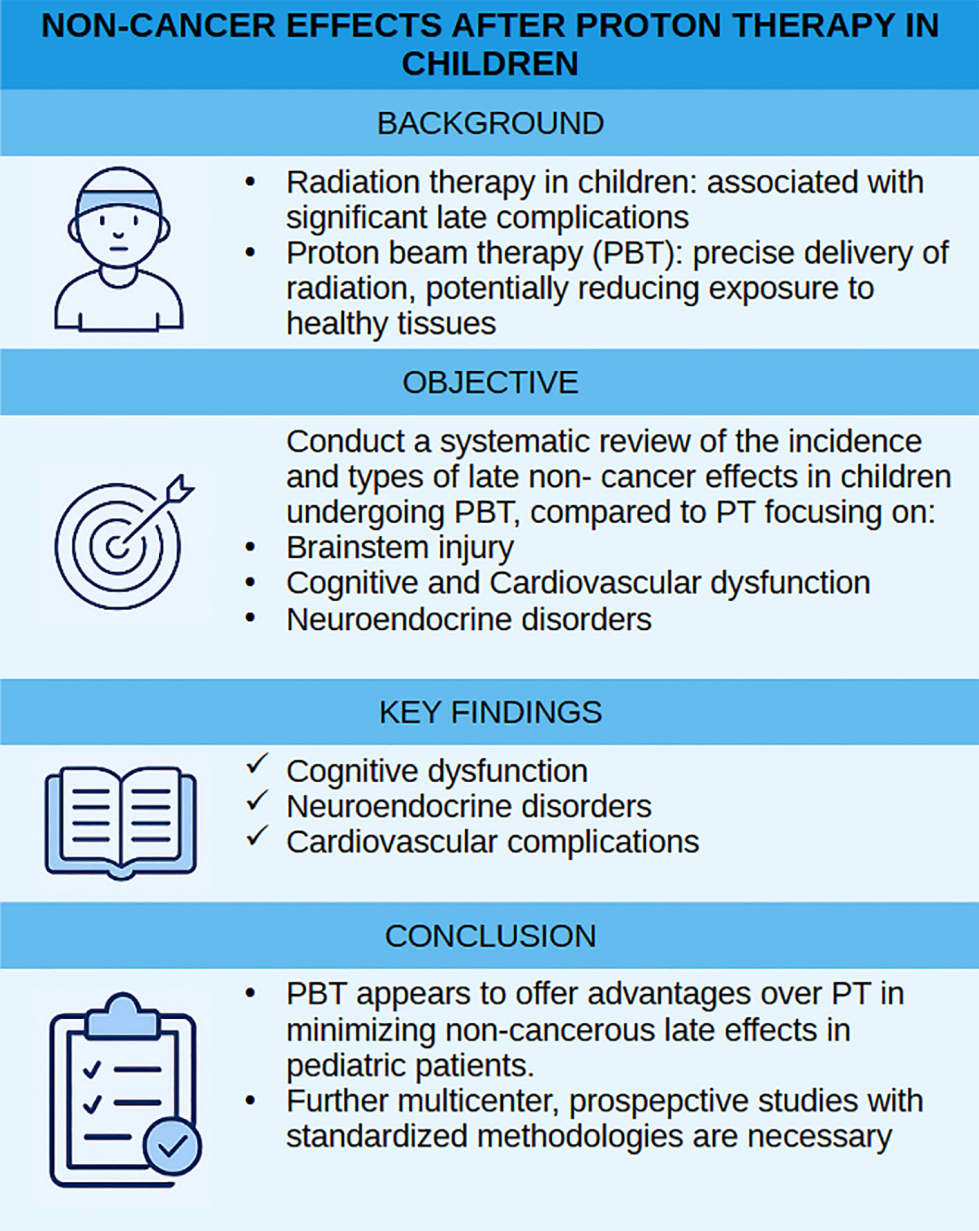
Summary figure.

In conclusion, while PBT appears to offer significant benefits in reducing late adverse effects, particularly among children, the current evidence base is not yet sufficient to draw definitive conclusions (**Figure 2**). A more coordinated, methodologically rigorous, and multidimensional research agenda is essential to fully understand and optimize the long-term safety and effectiveness of proton therapy. The risk of these non-cancer effects underscores the importance of long-term follow-up for children who undergo proton therapy. Regular monitoring and early interventions can help mitigate these impacts and improve the quality of life for pediatric patients. Therefore, to advance our understanding of the key determinants of non-cancer effects, further prospective studies are needed in large groups of patients, which will also allow for the analysis of factors related to proton radiotherapy.

## Glossary

AI: Adrenal Insufficiency
ATRT: Atypical Teratoid Rhabdoid Tumor
CCSS: Childhood Cancer Survivor Study
CI: Cumulative Incidence
CNS: Central Nervous System
CSI: Craniospinal Irradiation
CVD: Cardiovascular Disease
DBA: Daily Basic Activities
FSIQ: Full-Scale IQ
GHD: Growth Hormone Deficiency
HD: Hodgkin’s Disease
HL: Hodgkin Lymphoma
HPA: Hypothalamic-Pituitary Axis
HRQoL: Health-Related Quality Of Life;
IMRT: Intensity-Modulated Radiotherapy
INRT: Involved Node Radiotherapy
LET: Linear Energy Transfer
MB: Medulloblastoma
PBS: Pencil Beam Scanning
PBT: Proton Beam Therapy
PF: Posterior Fossa
PS: Passive Scattering
PT: Photon Therapy
QoL: Quality of Life
RBE: Relative Biological Effectiveness
RLAR: Risk of Lifetime Attributable Risk;
RR: Relative Risk
Radiotherapy
SHD: Sex Hormone Deficiency
SOBP: Spread-Out Bragg peak
3DCRT: Three-Dimensional Conformal Radiotherapy.
